# Energy-Efficient Multicast Service Delivery Exploiting Single Frequency Device-To-Device Communications in 5G New Radio Systems

**DOI:** 10.3390/s18072205

**Published:** 2018-07-09

**Authors:** Sara Pizzi, Federica Rinaldi, Antonella Molinaro, Antonio Iera, Giuseppe Araniti

**Affiliations:** DIIES Department, University Mediterranea of Reggio Calabria, 89100 Reggio Calabria, Italy; sara.pizzi@unirc.it (S.P.); federica.rinaldi@unirc.it (F.R.); antmolin@unirc.it (A.M.); antonio.iera@unirc.it (A.I.)

**Keywords:** 5G, New Radio, energy efficiency, device-to-device communication

## Abstract

The forthcoming fifth generation (5G) networks are claimed to deliver the large amount of traffic generated by the huge number of heterogeneous devices that constitute the Internet of Things (IoT). This unprecedented volume of both human- and machine-generated traffic to be managed imposes 5G network operators to move the focus from throughput-optimized to energy-efficiency-optimized resource allocation solutions. Device-to-device (D2D) communications are recognized as an effective offloading technique that the 5G network can exploit to boost the capacity and energy efficiency of future 5G networks. In this paper, we design a technique to efficiently deliver multicast traffic in a 5G New Radio (NR) network by exploiting the benefits of D2D communication and single-frequency operation in order to improve the overall network energy efficiency. In the designed solution, the subset of devices in better channel conditions are served through a conventional multicast transmission, while cell-edge devices receive the multicast service from relay nodes that simultaneously transmit in D2D mode the same content. The dimension of the multicast serving area and the set of D2D connections to establish are chosen in order to maximize the overall network energy efficiency. Performed simulation results show the effectiveness of the proposed solution under varying frame configurations and number of multicast devices.

## 1. Introduction

While the previous mobile technology generations (from 2G to 4G) focused on optimizing the performance of Human-to-Human communications in terms of coverage, bandwidth, and latency, the next generation mobile network (5G) moves the focus from Humans to Machines, since an unprecedented number of objects will join the Internet together with people. Indeed, the Internet of Things (IoT) forecast predicts that more than 50 billion devices will be connected by 2020 [1]. 5G application scenarios are characterized by the coexistence of both *human-based* (i.e., mobile video and high-QoE services) and *machine-based* (i.e., smart environments, intelligent transport systems, and software/firmware upgrade) services [2]. Furthermore, next generation access technologies will also support other kinds of services, such as Multimedia Broadcast/Multicast Service, location/positioning service, Vehicle-to-Everything (V2X) communications, and critical communication services. The latter are classified in public safety communications, emergency communications, and public warning/emergency alert systems [3]. In order to effectively support these additional services for 2020 and beyond, the International Telecommunication Union (ITU) [4] has accordingly defined three families of usage scenarios:

- enhanced Mobile Broadband (eMBB) coping with the significant increase of data volumes, overall data capacity, and user density;
- massive Machine Type Communications (mMTC) requiring low power consumption for a huge number of connected devices;
- Ultra-Reliable and Low Latency Communications (URLLC) providing safety-critical and mission critical applications.

The service heterogeneity imposed by the need to effectively manage the coexistence of Human-Type Communication (HTC) and Machine-Type Communication (MTC) requires that the 5G technology puts in place appropriate changes and improvements, at both application and customer levels, to manage resources efficiently at higher data-rates, lower latency and higher reliability, and to support effectively a huge number of connected devices.

MTC [5], also known as Machine-to-Machine communication (M2M), refers to data exchange among MTC devices and foresees a wide range of applications and services. Generally, MTC devices require high power consumption during a wireless communication. Thus, energy efficiency represents a very important concern in 5G networks that foresee the presence of a massive number of devices (i.e., sensor nodes) deployed for gathering environmental information, health-care monitoring, improving security, and so on.

5G shifts the focus on energy efficiency rather than throughput. That is why network operators are working hard with the aim to offer more and more efficient solutions in energy for 5G New Radio (NR) systems. The change of the point of view, from throughput-optimized to energy-efficiency-optimized solutions, is due to several reasons. On one hand, 5G has to face to the management of the wide and always-increasing diffusion of low-power MTC devices (i.e., sensor/machine nodes). Indeed, energy efficiency is a decisive feature for MTC devices, for example, when sensing and monitoring in disperse areas where it is difficult to regularly replace the battery. On the other side, the problem of the environment impact became more and more serious in the last decade. In particular, the Information and Communications Technology (ICT) industry is also responsible of the global power consumption by generating a hundred million tons of ${\mathrm{CO}}_{2}$. For these reasons, one of the main bets of 5G is to reduce energy consumption by ensuring longer battery life of sensor/machine nodes. By doing so, the forthcoming next generation technology not only aims to enhance the overall network performance, but it will also ensure a sustainable environmental development and to boost the system autonomy. Hence, Energy-Efficiency has actually become a key performance indicator for future 5G networks.

In order to enhance the network capacity and to boost the energy efficiency of 5G networks, offloading techniques, i.e., Device-to-Device (D2D) [6] communications, could be the key. D2D communications allow devices in close proximity to activate side-links (D2D links) by exploiting cellular radio resources. Supporting direct communications among neighboring devices reduces the cell-edge effect and increases the coverage extension, the Aggregate Data Rate (ADR), and the system energy efficiency, thanks to the lower transmit power compared to that needed for communication to the gNodeB (the new 5G base station).

Despite optimizing energy efficiency and exploiting D2D communications are two key topics in the forthcoming 5G network, significant work is still needed in order to increase the global energy efficiency while integrating the D2D paradigm in the traditional cellular communication.

According to the classification proposed in [7], the approaches for increasing the energy efficiency of wireless networks can be grouped under four categories: (i) resource allocation, (ii) network planning and deployment, (iii) energy harvesting and transfer, and (iv) hardware solutions. In this paper, we opt for the first category and propose Energy Efficient D2D over Single Frequency Network (hereinafter referred to as EED2D-SF), a resource allocation algorithm for multicast service delivery that couples conventional multicast with D2D communication and takes advantage from the Single Frequency paradigm to increase the network energy efficiency. The proposed scheme aims to face the challenging energy consumption problem of the current machine-type cellular communication, i.e., the LTE-M standard. The study considers all foreseen 5G frame configurations [8] that differ from current cellular frame configurations in the number and position of downlink and uplink slots. An overall simulation analysis is carried out with the aim to assess the performance of the proposed solution when focusing on file downloading (e.g., software upgrade/update), as one of the key use cases for the mMTC scenario.

The remaining of the paper is organized as follows. In Section 2, a brief summary of the main related work in the field is presented. Section 3 shows the reference system model. The proposed EED2D-SF scheme is described in Section 4, while performance evaluation results are discussed in Section 5. Conclusive remarks are reported in Section 6.

## 2. Related Work and Background

It is now a fact that multicast has significant potential to push the limits of next generation communication systems [9]. However, still several challenges have to be addressed. Among these, the design of effective radio resource management (RRM) policies is a critical point.

In Orthogonal Frequency Division Multiple Access (OFDMA) systems, the Adaptive Modulation and Coding (AMC) mechanism is commonly used; it appropriately chooses the transmission parameters in accordance to the channel conditions perceived on each link towards the base station. In particular, the Modulation and Coding Scheme (MCS) is set in order to cope with the user’s channel status (i.e., Channel Quality Indicator, CQI). In case of multicast services, transmission parameters must be selected by the base station on a per-group basis rather than on a per-user basis. It is highly probable that multicast group members measure different radio link qualities and, thus, require different MCSs. While poor links need robust MCSs to face hostile propagation conditions, users in favorable channel conditions could receive data at higher bit rates.

The RRM procedures designed for data multicasting can be classified in three macro-categories: conservative, opportunistic, and subgroup-based. The main example of conservative approach is the Conventional Multicast Scheme (CMS) [10], where all users receive the desired multicast content with the MCS level required for the correct reception of the user in the worst channel conditions. Therefore, users with a good channel quality suffer from low data rate. To overcome this inefficiency, the Opportunistic Multicast Scheme (OMS) [11] transmits the content to only the users with the best channel every Transmission Time Interval (TTI) in order to maximize the overall system throughput. Although OMS better utilizes the spectrum, it suffers from poor short-term fairness. In order to ensure a trade-off between throughput and fairness, the Multicast Subgrouping (MS) is proposed in [12]. The main idea behind MS is to group multicast users with similar channel qualities and perform a per-subgroup resource allocation.

D2D communications represent an innovative solution to improve the performance of users with low channel quality. Indeed, the D2D technology would offer the 5G system its capability of improving spectrum efficiency and throughput, reducing power consumption, enhancing cell coverage, and offloading the network. As we proposed in [13], high-performing D2D links allow some devices to forward a given video content, previously received by the cellular network, to cell-edge devices, thus improving their perceived quality. In particular, a D2D-enhanced CMS with Single Frequency (D2D-SF) is proposed with the aim to combine the use of CMS and D2D links for increasing the system aggregate data rate while preserving the short-term fairness, typically guaranteed by CMS.

Besides increasing the overall throughput, D2D communications are essential for reducing the energy consumption. Currently, energy consumption has become a major concern in the design of 5G wireless network. In the literature, several works offer solutions for reducing the devices’ energy consumption, with a consequent increase in the energy efficiency of the whole network. The energy efficiency improvement is achieved by following different approaches. In [14] a D2D-based solution is proposed that guarantees a trade-off between energy and spectral efficiency, with the aim to avoid co-channel interference between D2D and cellular links and to increase the devices’ battery life. The authors model the radio resource problem as a non-cooperative game and implement a resource allocation algorithm that satisfies spectral efficiency and transmission power constraints. The energy consumption issue of D2D communication is also treated in [15], where the authors propose a three-device topology (i.e., source, relay, destination) and exploit the data entropy (i.e., the data to be forwarded by the relays) for reducing the energy consumption. Through mathematical calculations, it is shown that including a relay, as an intermediate device between the source and the destination, brings energy efficiency advantages with respect to the direct source-to-destination communication. In summary, authors in [14,15] find solutions to improve the energy efficiency of D2D communications without considering the cellular network. Another work aiming at the energy maximization of D2D communications is [16] that proposes a power control based on the users’ channel conditions. Similarly to [15], the presence of relays is encouraged with respect to the single hop D2D direct communication. In this perspective, an improvement is discussed in [17], where an adaptive forwarding approach is proposed with the aim to choose the best relay devices which provide the greater energy efficiency in multi-hop D2D communications. Authors in [18] formulate the problem of energy consumption minimization as a convex optimization problem, and find an optimal solution for the transmission mode selection (macro cellular, D2D, or small cell cellular). In particular, the threshold is determined beyond which the D2D mode activation is preferable rather than the macro or the small cell modes. In [19], high energy efficiency is achieved in mmWave small cells through a multicast scheduling scheme that adjusts the D2D transmission power following a power control algorithm.

In the literature, particular interest has been gained by Energy efficiency in D2D-aided multicast service, above all in the last years. In [20], the authors propose a device-to-device multicast (D2MD) scheme to enhance the performance of content sharing by exploiting D2D communication established on the basis of the social relationship and the physical distance among users. Furthermore, the scheme also proposes resource allocation with the aim to maximize the throughput and to overcome the problem of interference by performing power control and channel assignment. The social relationship and the physical distance among users are also taken into account in [21], where the energy efficiency of D2D communications is maximized by properly selecting the optimal channel mode and the transmission power to each user with an adaptive genetic algorithm. Diversely from [20], the proposed solution in [21] does not consider multicast transmissions. In [22] D2D communications are exploited for the retransmission of data, received in multicast from the Base Station (BS), by using the same channel (rather than multiple channels) in TDMA mode. The scheme minimizes the energy consumption of retransmitters by optimizing their transmission power. Another work dealing with D2D multicasting is [23], where the authors formulate a heuristic gradient power allocation algorithm to solve a resource allocation problem in order to maximize the energy efficiency of multicast D2D communications with the aim to satisfy the constraints of spectral efficiency and maximum transmission power.

Nevertheless, in the literature most of the works deal with D2D in the case of HTC. Instead, MTC-D2D and the evaluation of energy efficiency in a MTC scenario are little investigated topics. Indeed, to the best of our knowledge, an in-depth analysis is still missing on D2D-aided group-oriented service (i.e., software upgrade/update) delivery to MTC devices over 5G NR networks, which is the topic of this paper. Differently from [13], the proposed EED2D-SF aims to improve the overall network (not only D2D) energy efficiency with a new resource allocation technique. The analysis of the network energy efficiency is performed under different cellular frame configurations, which has not been done before. Furthermore, we assume that: (i) D2D communications exploit uplink resources, and unused downlink resources are destined to other kinds of requests, and (ii) forwarding devices in charge of relaying the received data to D2D receivers operate in Single Frequency mode. By summarizing, with respect to the literature in the field, our proposed Energy-Efficient D2D over Single Frequency Network scheme (named as EED2D-SF) introduces the following contributes:

- Focuses on a mMTC (rather than on a human-oriented) scenario where sensor/machine nodes are interested in file downloading, one of the key use cases for such a scenario.
- Maximizes the network energy efficiency, as it represents a key requirement for the analysed scenario composed by a huge number of energy-constrained nodes.
- Exploits D2D communication to both reduce the energy consumption and increase the system data rate by properly setting the multicast-area transmission and D2D communications from appropriately selected relay nodes.
- Avoids interference between cellular operation and D2D communications by exploiting downlink subframes for the transmission from the gNodeB to the multicast receivers, and uplink subframes for data forwarding from selected relays to cell-edge devices.
- Takes advantage from the single frequency operation for D2D communications in order to avoid interference among D2D groups without the need to perform power control and channel assignment.
- Analyses all 5G frame configurations in the performance analysis.

## 3. System Model

NR [24] is the new OFDM-based air interface designed for 5G systems. Orthogonal Multiple Access (OMA) is the NR basic multiple access scheme for both downlink and uplink data transmissions, where time and frequency physical resources of different users/devices do not overlap.

NR supports optimized OFDM-based waveforms, thanks to scalable numerology and Transmission Time Interval (TTI). This brings to different values of Subcarrier Spacing (SCS), scaling from 15 to 480 KHz. As highlighted in Equation (1), the SCS Δf values depend on the numerology μ.

(1) $$\Delta f=15KHz\times 2^{\mu }$$

Downlink and uplink transmissions are organized in frames. Each frame consists of 10 subframes, each lasting at maximum 1 ms. With the introduction of SCS, the number of slots in a frame depends on the numerology, and a slot can last from 31.25 μs to 1 ms. More details are shown in Table 1. A slot is 14 OFDM symbols with 61 available configurations [24]. OFDM symbols in a slot can be classified as *downlink* (denoted D), *flexible* (denoted X), or *uplink* (denoted U). In a slot in a downlink frame, downlink transmissions only occur in *D* or *X* symbols. In a slot in an uplink frame, uplink transmissions only occur in *U* or *X* symbols.

NR is designed to support a wide variety of 5G services and devices, deployments and spectrum ranges. The available radio spectrum is managed in terms of Resource Blocks (RBs). In the frequency domain, each RB corresponds to 12 consecutive and equally spaced subcarriers. Differently from Long Term Evolution (LTE), where the subcarrier spacing is 15 KHz and a RB spans 180 KHz (15 KHz × 12), in NR the RB bandwidth varies with the numerology (i.e., with μ=0, RB = 180 KHz; with μ=1, RB = 360 KHz; with μ=2, RB = 720 KHz; and so on).

In NR, the new base station is named as next-generation Node B (simply gNodeB or gNB). Each MTC device or sensor/machine node transmits its channel quality indicator (CQI) to the gNodeB that performs scheduling procedures, by setting the MCS transmission parameters and assigning radio resources to the scheduled MTC device at every TTI.

Each MTC device can support two transmission modes: the *cellular mode*, when the MTC device communicates through the gNodeB; and the *D2D mode*, when the MTC device bypasses the gNodeB and exploits direct connections (D2D link or sidelink). In this paper, we assume that D2D communications take place in the uplink subframe. This assumption guarantees a more efficient radio resources reuse [25]. By doing so, some downlink resources are destined for other services (i.e., unicast requests). The performance of the proposed EED2D-SF scheme is also affected by the considered frame configuration, since each configuration implies a different number and position of Uplink and Downlink subframes within the frame.

## 4. Energy Efficient D2D over Single Frequency Network (EED2D-SF)

In this work, we design a technique to be implemented in the gNodeB of a 5G network to efficiently deliver multicast traffic by exploiting the benefits of D2D communications and SFN operation. The aim is to improve the overall energy efficiency of the network. In particular, the subset of devices in better channel conditions are served by the gNodeB through a multicast transmission according to the traditional CMS approach, while devices located far from the gNodeB receive the multicast content via D2D communication by relay nodes, properly selected among those that received the multicast service. The relay nodes behave according to the SFN operation, i.e., they simultaneously transmit the same content to the D2D receivers.

The exploitation of D2D communications for providing service to cell-edge devices both improves the network data rate and reduces the energy consumption. Proximity connections between relay nodes and devices located close to the cell border will likely benefit from good channel conditions. Furthermore, as a consequence, the CMS serving area will be reduced, thus allowing the gNodeB to serve multicast devices with a less robust MCS that achieves higher data rates.

The signal quality received by the D2D terminals is improved thanks to the Single Frequency paradigm. In fact, the transmitting relay nodes in the cell are synchronized and simultaneously send the same signal received from the gNodeB, over the same uplink frequency to their D2D receivers. The latter observe multiple delayed versions of the same signal and, through appropriate synchronization, channel estimation, and equalization techniques, benefit from the multipath diversity at the only cost of a slight increase in the computational complexity. The dimension of the CMS serving area (i.e., the choice of the MCS for the multicast transmission) and the set of D2D connections to establish (i.e., the choice of the MCS for D2D transmissions) are chosen by the gNodeB in order to maximize the overall network energy efficiency.

**Figure 1:**
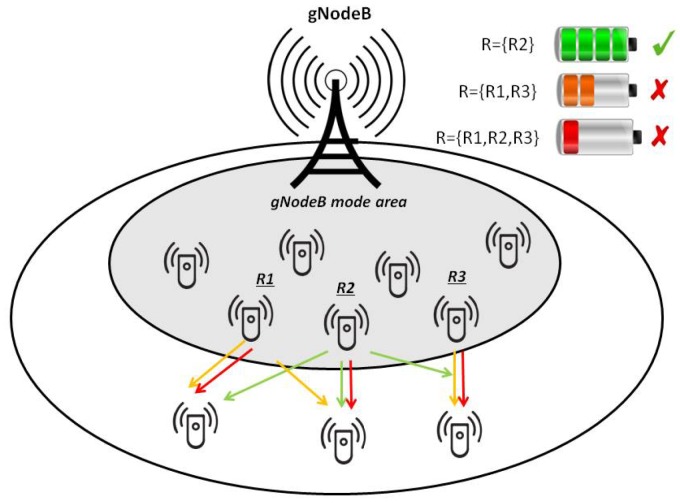
Energy Efficient D2D over Single Frequency Network (EED2D-SF) scenario.

Figure 1 shows the scenario considered in this paper. After receiving data from the gNodeB, some devices acting as relay nodes (i.e., R1, R2 and R3) could forward the stored data to nodes left out from the gNodeB transmission. Three possible relay selection options with the related D2D configurations are shown (represented with different colors) corresponding to three levels of energy consumption. The first configuration, depicted with the red color, selects three relays, R1, R2 and R3; the second configuration, with orange links, exploits two relays, R1 and R3; and the third configuration selects only one relay, R2, and is illustrated with green links. The relays simultaneously forward data to cell-edge devices. As can be seen, each configuration is identified by a color according to the overall network energy consumption level. Specifically, the most energy efficient D2D configuration is represented by the green battery, the worst performing D2D configuration in terms of energy efficiency is represented by the red battery, and the D2D configuration with the orange battery is characterized by an intermediate energy consumption level. Our proposed EED2D-SF will select the configuration identified with the green color, since it exhibits the best performance in terms of overall network energy efficiency because of both the number of devices involved and the position of cell-edge devices with respect to the relay node.

Various energy efficiency (EE) Key Performance Indicators (KPIs) are defined by Standard Development Organization (SDOs). They can be applied to the whole network (i.e., end-to-end), sub-networks (e.g., the radio access network), single network elements, or to telecommunication sites, which contain network elements and site equipment. In [26] EE metrics and measurement procedures in operational radio access networks are defined.

The EE KPI we will refer in this work is the Mobile Network data Energy Efficiency ($EE_{MN,DV}$), expressed in bit/J, and defined as the ratio between the data volume $DV_{MN}$ and the energy consumption ($EC_{MN}$):

(2) $$EE_{MN,DV}=\frac{DV_{MN}}{EC_{MN}}$$

In practice, it is the amount of information that can be reliably transmitted per Joule of consumed energy. Starting from this performance metric, the gNodeB determines the MCSs for the multicast and the D2D transmissions that maximize the overall Network Energy Efficiency (NEE), as in the following:

(3) $$\underset{\left(CQI_{CMS},CQI_{D2D}\right)}{\mathrm{arg max}}\left\{\sum_{i=1}^{n_{CMS}}\frac{DV}{EC_{i,RX}^{CQI_{CMS}}}+\sum_{j=1}^{n_{D2D}}\frac{DV}{EC_{i,RX}^{CQI_{D2D}}}+\sum_{k=1}^{n_{rel}}\frac{DV}{EC_{k,TX}^{CQI_{D2D}}}\right\}$$

where the three sums represent the EE of, respectively, the $n_{CMS}$ devices receiving the multicast content DV by using the $CQI_{CMS}$ MCS; the $n_{D2D}$ devices receiving the same multicast content by relay nodes in D2D mode using the $CQI_{D2D}$ MCS; and the $n_{rel}$ relay nodes that forward the multicast content to D2D receivers by using the $CQI_{D2D}$ MCS.

Equation (3) is subject to:

(4) $$N_{gNB}\cup N_{D2D}=N$$

where N is the total number of MTC devices, $N_{gNB}$ is the number of MTC devices served through gNodeB transmission and $N_{D2D}$ is the number of MTC devices receiving data in D2D communications.

**Figure 2:**
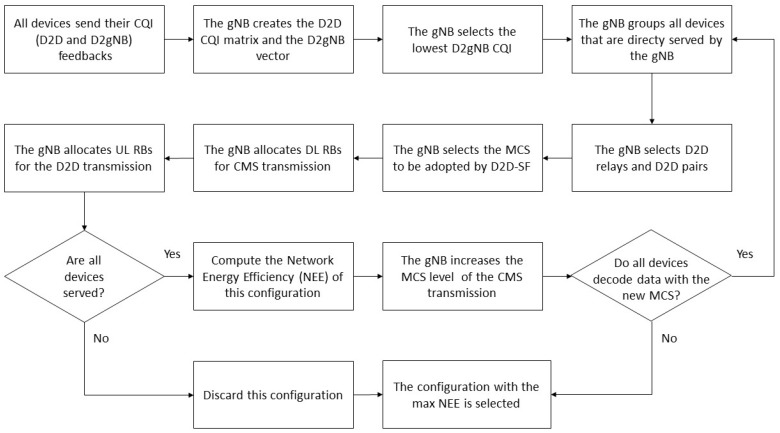
EED2D-SF flow chart.

The EED2D-SF follows an iterative approach analyzing all possible combinations of multicast (from gNodeB to relays) and D2D (from relays to cell-edge devices) connections, and follows the steps shown in the flow chart in Figure 2. In particular, the gNodeB receives CQI feedbacks from all devices regarding both the device-to-gNodeB (D2gNB) and the D2D links. The D2gNB CQIs are candidates as potential CQIs for the CMS in the downlink subframes. Then, starting from the lowest device-to-gNodeB CQI and increasing it at each iteration, the gNodeB determines the subgroup of devices that can receive data via multicast directly from the gNodeB for the considered CQI. The selected multicast devices at a given iteration are those that, according to their channel conditions (the CQI under investigation), are able to receive data sent from the gNodeB with the currently selected MCS. Then, a subset of these nodes must be selected as relays towards the devices out-of-coverage from the multicast transmission. Specifically, the relay nodes will be those allowing to serve the highest number of out-of-multicast-coverage sensor/machine nodes. Then, the MCS to be used for D2D communication is chosen as the most robust among those supported by the selected relay nodes, in order to exploit the single frequency operation. If all devices are served through either CMS or D2D transmissions, the algorithm computes and stores the achievable NEE value as shown in Equation (3) for the current configuration; otherwise, the configuration is discarded. The algorithm stops when all configurations have been analyzed and the gNodeB selects the one that guarantees the highest overall network energy efficiency and provides service to all devices.

The implementation of the proposed EED2D-SF algorithm has an overall complexity equal to $O(N^{2})$, where N is the number of MTC devices. We highlight that the designed scheme is executed at the gNodeB that is a high performing node. Hence, the implementation of the proposed scheme is feasible.

## 5. Performance Evaluation

### 5.1. Simulation Model

Simulation campaigns have been conducted in MATLAB, by following the guidelines in [27,28], in order to validate the capability of the proposed EED2D-SF scheme to perform a network resources allocation that is able to minimize the network energy consumption while also being efficient in terms of delivered data rate to devices. We concentrated our attention on a mMTC scenario (1000 sensor/machine nodes located inside a (1000 m × 1000 m) area) where the gNodeB is in charge of delivering to all devices a software update (10 MBytes).

The main simulation parameters, listed in Table 2, have been set according to [29]. We have considered that R = 100 RBs are available in the system on a 20 MHz channel bandwidth. Channel conditions for the MTC devices are evaluated in terms of signal-to-interference-plus-noise ratio (SINR) experienced on each sub-carrier when the path loss and fading phenomena affect the signal reception. The effective SINR is mapped onto the CQI level that ensures a block error rate (BLER) smaller than 1%. In particular, 15 CQI levels are considered. We set the scheduling frame duration to 10 ms. During this 1s-long session, 100 data frames are transmitted, with 10 TTIs per data frame, and each TTI lasting 1ms as we set μ=0 (sub-carrier spacing equal to 15 KHz). According to [30], we set the gNodeB and D2D node transmission power to 46 dBm and 23 dBm, respectively. In addition, the noise spectral density is equal to −174 dBm/Hz [30]. The propagation model is described by the pathloss formula *L* = 128.1 + 37.6*log(*d*) [30], where *d* is the distance (expressed in Km) between the gNodeB and the sensor/machine node. Finally, the shadowing standard deviation is set to 10 and 12 dB in cellular and D2D mode, respectively [30].

The power consumption values are set as in [31]. Specifically, the power consumption of device *i* in the cellular network is:

(5) $$P_{c_{i}}=\beta_{cellular}+\alpha_{d}\cdot R_{c_{i}}$$

where $\beta_{cellular}$ is the baseline power, i.e., 1288.04 mW [31], $\alpha_{d}$ is the downlink power consumption, i.e., 51.97 mw/Mbps [31], and $R_{c_{i}}$ is the downlink data rate (in Mbps) for sensor/machine node *i* over the cellular interface, which depends on the allocated RBs and the channel quality experienced by the sensor/machine node.

A variable number of MTC devices is uniformly distributed in the gNodeB coverage area. We assume that, every TTI, all MTC devices send a CQI feedback about the channel condition perceived on the link towards the gNodeB. The CQIs are mapped onto an MCS level that ensures a Block Error Rate (BLER) smaller than 1%.

The simulations focus on evaluating the EED2D-SF performance when considering the case of file downloading (e.g., software update), one of the most interesting applications in the mMTC usage scenario. In particular, the study considers the delivery of a 10 MB file and evaluates the network performance by considering all the Time Division Duplexing (TDD) frame configurations [29] foreseen by the 5G NR standard (in Table 3).

The performance analysis has been conducted in the following two scenarios:

- **Scenario A**, simulating a dense network of devices, where we analyze the performance of the proposed EED2D-SF with respect to CMS and D2D-SF [13] under different TDD frame configurations. The number of MTC devices and the number of available radio resources (i.e., RBs) are set to 1000 and 100, respectively.
- **Scenario B**, where we analyze the performance of the proposed EED2D-SF for all the TDD frame configurations when varying the number of MTC devices from 200 to 1000, while the number of RBs is fixed to 100.

The following performance metrics have been considered:

- **Network Energy Efficiency (NEE)**, measured as the ratio between the downloaded file size and the energy consumption required for its reception/transmission;
- **Mean Throughput**, measured as the mean data rate achieved by the multicast members;
- **Delivery Time**, as the time needed for completely downloading the whole file;
- **Aggregate Data Rate (ADR)**, computed as the sum of the data rates achieved by the multicast MTC devices.

### 5.2. Results Analysis

**Figure 3:**
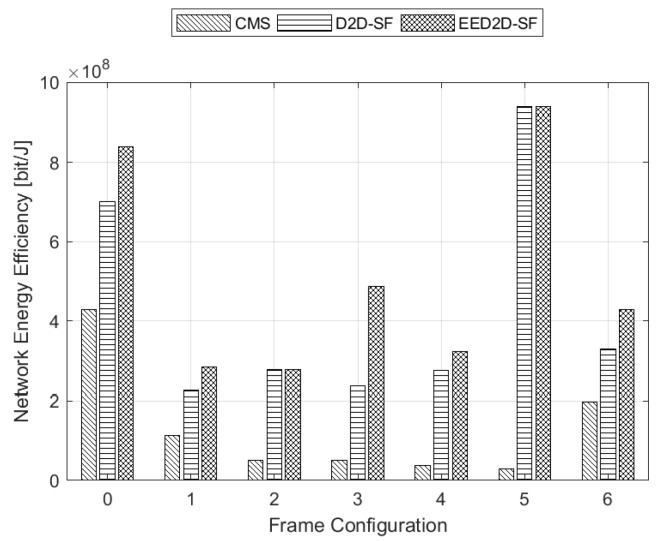
Network Energy Efficiency for all frame configurations.

We first analyze the results achieved under Scenario A. Figure 3 shows the NEE achieved by the analysed schemes under all frame configurations foreseen by the NR standard. We notice that the traditional CMS approach exhibits really poor performance with respect to the other schemes. Furthermore, CMS reaches the highest NEE value for the frame configuration with the lowest number of DL subframes (i.e., frame configuration No. 0). On the opposite, the most performing solution in terms of NEE, as desired, is our proposed EED2D-SF that presents the highest NEE value for the frame with the highest number of DL subframes (i.e., frame configuration No. 5). We also notice that when the number of UL subframes in the TDD frame is low (frame configurations No. 5 and 2), both EED2D-SF and D2D-SF algorithms converge to the same NEE values.

**Figure 4:**
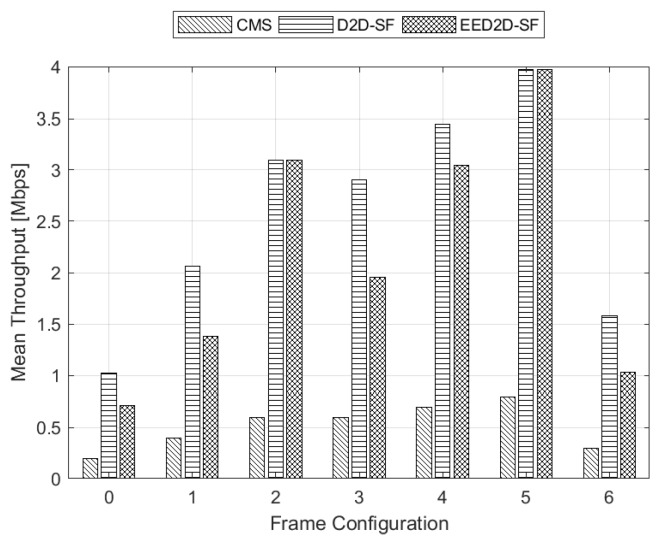
Mean Throughput for all frame configurations.

The motivation behind these results can be better understood when analyzing the performance in terms of mean throughput reported in Figure 4. Since CMS does not exploit UL subframes, there is an inverse proportionality between mean throughput and NEE. In fact, the higher the throughput, the lower the NEE. The same reasoning does not hold for the other algorithms (D2D-SF and EED2D-SF), since UL subframes are used to convey data towards D2D receivers. By looking at Figure 4 with respect to Figure 3, we appreciate that better results in terms of NEE are at the expense of a slight mean throughput reduction.

**Figure 5:**
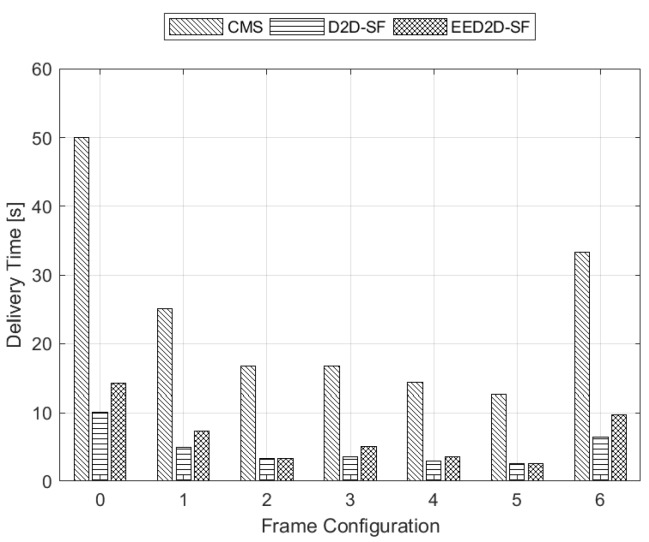
Delivery time for all frame configurations.

As expected, the higher the throughput, the lower the delivery time. Indeed, Figure 5 shows that frame configuration No. 5 offers the lowest delivery time, and it is classified as the frame configuration which achieves both the highest NEE and mean throughput in the shortest time interval.

**Figure 6:**
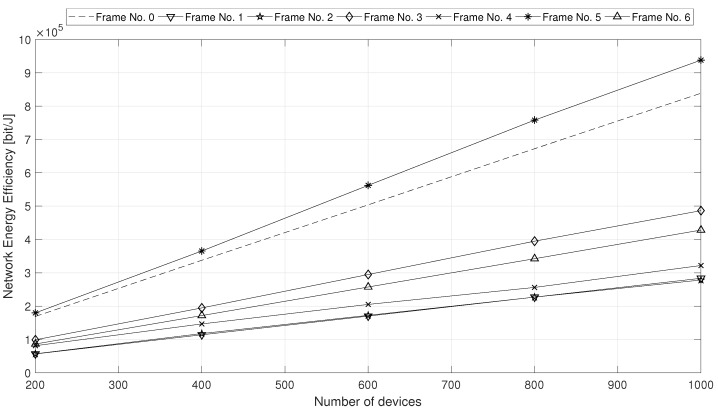
Network Energy Efficiency under increasing number of multicast MTC devices.

**Figure 7:**
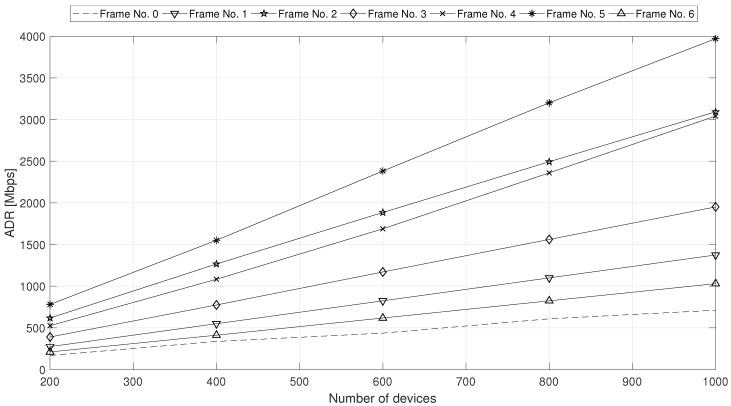
ADR under increasing number of multicast MTC devices.

From now on, we focus attention only on the performance of the proposed EED2D-SF scheme. Hence, we analyze the results obtained in scenario B, where we assess how EED2D-SF works under an increasing number of devices (which varies from 200 to 1000). We evaluate the performance with all TDD frame configurations by analyzing NEE (Figure 6) and ADR (Figure 7) metrics.

Both NEE and ADR exhibit a performance enhancement with the increase in the number of devices interested in the transmitted content. This is due to the cumulative nature of both metrics. More interesting is the substantially different result that can be achieved by considering the various frame configurations. Since the relay devices forward to their D2D receivers the data directly received from the gNodeB, the presence of a large number of DL subframes allows to buffer a high volume of data to be forwarded through D2D links during the UL subframes. Hence, the capacity of D2D links is better exploited, leading to higher mean throughput (and consequently ADR) values. For this reason, the frame configuration No. 5 is the most performing. On the opposite, the frame configuration No. 0 (with the highest number of UL subframes) does not exploit all the UL subframes for data forwarding via D2D communications and shows low ADR performance.

## 6. Conclusions

In this paper we proposed Energy Efficient D2D over Single Frequency Network (EED2D-SF), a resource allocation algorithm for multicast service delivery that couples the conventional multicast scheme with D2D communications. It exploits the Single Frequency concept for the D2D transmission, with the aim to increase the network energy efficiency in 5G New Radio Networks. A simulation campaign has been conducted in case of file downloading (i.e., software upgrade/update) in a mMTC scenario by taking into account all frame configurations foreseen by the 3GPP standard.

Simulative results demonstrate that, in a context where the energy consumption of mobile devices is an increasingly important concern, the proposed EED2D-SF is the most performing algorithm in terms of network energy efficiency with respect to other compared solutions.

An extremely interesting future direction of investigation is the analysis of a mobile scenario. In fact, as a future work we intend to analyze the feasibility of implementing the proposed solution in a V2X scenario.

## Figures and Tables

**Table 1:** NR numerology, Subcarrier Spacing, Number of OFDM symbols per slot, Number of slots per frame, Number of slots per subframe and Time Slot.

μ	Δf	No. Symbols Per Slot	No. Slots Per Frame	No. Slots Per Subframe	Time Slot
0	15 KHz	14	10	1	1000 μs
1	30 KHz	14	20	2	500 μs
2	60 KHz	14	40	4	250 μs
3	120 KHz	14	80	8	125 μs
4	240 KHz	14	160	16	62.5 μs
5	480 KHz	14	320	32	31.25 μs

**Table 2:** Simulation Parameters.

Parameter	Value
Cell size	1000 m × 1000 m
Number of MTC devices	1000
TTI	1 ms
μ	0
RB size	12 sub-carriers, 0.5 ms
Scheduling frame	10 ms
Sub-carrier spacing	15 kHz
eNodeB Tx power	46 dBm
D2D node Tx power	23 dBm
Noise spectral density	−174 dBm/Hz
Distance attenuation	128.1 + 37.6*log(*d*), *d* (km)
Shadowing standard deviation	10 dB (cell mode); 12 dB (D2D mode)
BLER target	1%

**Table 3:** TDD Frame Configurations (U: Uplink; D: Downlink) [29].

Configuration	Subframe Number									
	0	1	2	3	4	5	6	7	8	9
0	D	S	U	U	U	D	S	U	U	U
1	D	S	U	U	D	D	S	U	U	D
2	D	S	U	D	D	D	S	U	D	D
3	D	S	U	U	U	D	D	D	D	D
4	D	S	U	U	D	D	D	D	D	D
5	D	S	U	D	D	D	D	D	D	D
6	D	S	U	U	U	D	S	U	U	D

## Abbreviations

The following abbreviations are used in this manuscript:

IoT	Internet of Things
D2D	Device-to-Device
EE	Energy Efficiency
HTC	Human-Type Communication
MTC	Machine-Type Communication
QoE	Quality of Experience
eMBB	enhanced Mobile Broadband
mMTC	massive Machine Type Communication
URLLC	Ultra-Reliable Low Latency Communications
V2X	Vehicle-to-Everything
CMS	Conventional Multicast Scheme
MCS	Modulation and Coding Scheme
SFN	Single Frequency Network
NR	New Radio
SCS	Subcarrier Spacing
OMA	Orthogonal Multiple Access
OFDM	Orthogonal Frequency Division Multiplexing
TTI	Transmission Time Interval
CQI	Channel Quality Indicator
RB	Resource Block
KPI	Key Performance Indicator
BLER	BLock Error Rate
TDD	Time Division Duplexing
ADR	Aggregate Data Rate
NEE	Network Energy Efficiency
